# Effects of the Feed: Water Mixing Proportion on Diet Digestibility of Growing Pigs

**DOI:** 10.3390/ani9100791

**Published:** 2019-10-12

**Authors:** Cinta Sol, Lorena Castillejos, Sergi López-Vergé, Ramon Muns, Josep Gasa

**Affiliations:** 1 Animal Nutrition and Welfare Service, Department of Animal and Food Sciences, Universitat Autònoma de Barcelona, 08193 Bellaterra, Spain; cintasol.llop@gmail.com (C.S.); Sergio.Lopez.Verge@uab.cat (S.L.-V.); Ramon.Muns@afbini.gov.uk (R.M.); josep.gasa@uab.cat (J.G.); 2 Agri-Food and Biosciences Institute, Large Park, Hillsborough, Co. Down, Northern Ireland BT26 6DR, UK

**Keywords:** feed digestibility, swine, water-diluted diet, water-to-feed ratio

## Abstract

**Simple Summary:**

The effect of different water-to-feed ratios on apparent total tract digestibility was evaluated in growing–finishing pigs. Interest in giving pigs wet feed has increased in recent decades. However, there are still many concerns about doing it efficiently. In this study, the effects of different water-to-feed ratios on the digestibility of energy and nutrients in growing–finishing pigs were evaluated. Our results demonstrate that the optimal water-to-feed ratio to improve organic matter and gross energy digestibility varies depending on the age of the animal. In particular, the water-to-feed ratios that improve digestibility coefficients were lower for young growing pigs and higher for older finishing pigs. The data suggest that optimum efficiency is reached with a less water-diluted diet for young animals, when compared to older pigs.

**Abstract:**

The effect of different water-to-feed ratios on apparent total tract digestibility of energy and nutrients were evaluated in growing–finishing pigs. In trial 1 (26 d), 16 female pigs (46.7 ± 1.98 kg of body weight, BW) were individually assigned to four treatments (n = 4). In trial 1, pigs were fed a control diet in dry form (CON) and in blend form with water-to-feed ratios of 0.6:1, 2.1:1 and 2.7:1. In trial 2 (26 d), rearranged animals (65.4 ± 3.14 kg of BW) were assigned a control diet in dry form (CON) and ratios of 1.35:1, 2.7:1 and 3.5:1. In trial 1, pigs fed on ratios of 2.1:1 and 2.7:1 had a higher organic matter digestibility (OMd) and gross energy digestibility (GEd) than CON. In trial 2, pigs fed on ratios of 1.35:1, 2.7:1, and 3.5:1 had a higher OMd and GEd than CON. Quadratic regressions showed the maximum dilution rate to reach higher digestibility coefficients at 1.83:1 and at 2.7:1 for trials 1 and 2, respectively. During trial 1, pigs on the 0.6:1 dilution rate had higher weight gain than those on CON. The water-to-feed ratio that optimized OM and GE digestibility may increase with the age of the pigs.

## 1. Introduction

Liquid feed has become a popular feeding system for pigs in many European countries. It consists of a blend compound feed (mixture of raw materials “as fed”) with water or liquid food-industry co-products, in a central tank in the pig unit, before delivery through a pump-and-pipes mechanical system [1,2]. Liquid feed contains about 700–800 g of water per kg, since the capacity of the pump-and-pipes devices determines the upper limit for dry matter in the mixture. Liquid feeding should not be confused with either fermented liquid feeding (see paper of Canibe and Jensen [2]) or wet/dry feeding systems, which we define as any kind of feeder device which also includes a water supply, usually as a nipple. In the wet/dry system, water and the compound feed are kept separate up to the point of delivery to the pig. The main difference between liquid and wet/dry systems is the water-to-feed ratio and homogeneity of the mixture, which is usually higher for liquid feeding, and the period of time that the compound feed is in a liquid medium before it is consumed by the pig, which is usually lower in the wet/dry system. This may have important implications for nutrient digestibility, feed intake, and performance [1,3].

The optimum dry matter content of liquid feed depends on the age of the pigs, feed composition, environmental conditions and water quality [4].

In piglets, during the first few weeks after weaning, liquid feeding at a 2.5:1 water-to-feed ratio increased post-weaning growth rates compared to a 3.5:1 ratio [5]. In contrast, at higher ratios (>4:1), digestible energy intake is depressed [6]. In growing–finishing pigs, the water-to-feed ratio had no significant effect on feed intake [7]. However, BW gain and feed conversion significantly improved as the water content of the liquid feed was increased (from 2.0:1 to 3.5:1) [7]. Similar results were found by Barber et al. [8] when the ratio ranged from 1.63:1 to 3.25:1, and the digestibility coefficient also increased from 0.791 to 0.829. 

The aim of the present study was to evaluate the effect of different dilution ratios of water:mixed feed on growing–finishing pigs of two different ages, focusing on the feed digestibility of energy and nutrients.

## 2. Materials and Methods

The experiment was performed at the animal research facilities, Servei de Granges i Camps Experimentals, of the Universitat Autònoma de Barcelona, Spain. The present work was conducted under the approval of the Animal Ethics Committee of the Universitat Autònoma de Barcelona and complied with European Union guidelines for the care and use of research animals.

### 2.1. Experimental Design, Animals, Housing and Diets

Sixteen females (Landrace × Large White), weighing 46.7 ± 1.98 kg of BW, were used and randomly allotted, according to BW, one of four dietary treatments (4 pens/treatment and 1 pig/pen). The total experiment lasted 52 days and was divided into two trials consisting of 26 days per experiment. When finishing the first trial, pigs weighing 65.4 ± 3.14 kg of BW were randomly distributed again among the second trial treatments. The initial 20 days of each trial were considered an adaptation period to the diet. During this period, the feed was delivered in a semi-ad libitum manner, increasing or decreasing the amount of feed offered daily from 3% to 5%, depending on the previous day’s registered refusals. The diet was always offered twice a day in equal meals (09:00 h and 16:00 h). Following the adaptation period, the daily amount of feed on offer per pig was maintained until the end of the trial, Day 26. Feed refusals were collected daily, and dried and weighed to calculate feed intake.

In trial 1, the diet was offered as a dry diet (“as fed”) for the control diet (CON), and as three wet or liquid mixtures produced by mixing tap water and feed in ratios of 0.6:1 (0.6 parts of water per one part of feed), 2.1:1, and 2.7:1, respectively. The blend of compound feed with water was made by manually mixing water and dry feed fifteen minutes before feeding to avoid fermentation. In trial 2, the diet was offered again as a dry diet (CON), and mixed with water in ratios of 1.35:1, 2.7:1, and 3.5:1, respectively. The water-diluted diets were made in the same way as in trial 1. The control diet (CON) and 2.7:1 ratio were maintained throughout the whole experiment. 

The ingredients and the chemical composition of the dry diet are summarized in Table 1. The feed was manufactured in dry mash form and was formulated to meet or slightly exceed the FEDNA [9] nutrient requirements.

Each pen was equipped with a one-sided, stainless steel feeder and a nipple drinker to guarantee free access to water throughout the experimental trial. After each meal, the feeders were cleaned and feed refusals removed to avoid possible fermentation. All pigs were housed in an environmentally controlled room.

### 2.2. Sampling and Measurements

Individual pig BW was recorded at the beginning and the end (Day 26 in trial 1 and Day 52 in trial 2) of the experiment to determine average daily gain (ADG). Feed consumption was recorded every day by weighing and drying the leftover in order to calculate average daily feed intake (ADFI). 

Apparent total tract digestibility of organic matter (OMd), ether extract (EEd), crude protein (CPd), crude fiber (CFd), and gross energy (GEd) were determined using titanium dioxide as an indigestible marker. Titanium dioxide was included (3 g/kg) in the dry feed throughout the experiment. Fresh fecal grab samples were collected from all pigs twice a day on Days 25 and 26 in trial 1 and twice a day on Days 51 and 52 in trial 2, via rectal massage. 

### 2.3. Chemical Analysis and Calculations

All fecal samples were dried at 65 °C for 96 h in a forced-air oven, mixed and pooled until analysis. Before chemical analysis, the fecal samples were defrosted, after which they were finely ground to a size that could pass through a 1 mm sieve. All feed samples were collected at the beginning of each trial, and the average value of the analyzed composition was used to represent feed composition and calculate the digestibility coefficients. 

All feed and fecal samples were analyzed for dry matter (DM), ash, gross energy (GE), ether extract (EE), crude protein (CP), and crude fiber (CF) following the procedures outlined by the Association of Official Analytical Chemists [10]. The DM was determined on an aliquot sample to establish the residual water content after drying for 24 h at 103 °C and the ash content was determined after ignition of a weighed sample in a muffle furnace (Carbolite CWF 1100, England, Hope Valley) at 550 °C for 6 h. The corresponding analytical result was expressed on a DM basis. The GE content of diets and fecal samples was determined using an oxygen bomb calorimeter (IKA – Calorimeter system C 4000 Adiabatic, Staufen, Germany). The EE of diets and feces was analyzed by a solvent extraction system (Soxtec™ 2055 FOSS, Höganäs, Sweden). The CF of diets and feces was analyzed using the Ankom 220 Fibre Analyser Unit (ANKOM Technology Corporation; Macedon, NY, USA). The CP of diets and feces was determined by the Kjeldhal system (Kjeltec 8400 Analyzer Unit FOSS, Höganäs, Sweden). The CP was determined as total N × 6.25. 

Titanium dioxide was analyzed via UV absorption spectrophotometry, following the method described by Short et al. [11].

The in vivo digestibility of DM, GE, and nutrients was calculated from the difference between nutrients in the feed and nutrients in the feces, after correction by the indigestible marker.

### 2.4. Statistical Analysis

Data were analyzed by a one-way ANOVA using the GLM procedure of SAS (SAS 9.3 version, SAS Institute INC.; Cary, NC, USA) and results are presented as LS means. Differences between groups were assessed using the Tukey test. The pig was the experimental unit for all the variables studied.

A regression analysis was performed using the REG procedure of SAS, to show the effect of the dilution ratio on digestibility coefficients and growth performance parameters.

Finally, in all statistical analyses, significant differences were declared at *p* ≤ 0.05, while 0.05 < *p* ≤ 0.10 were considered to indicate a tendency.

## 3. Results

Throughout the experiment, all pigs remained healthy, showed normal behavior and willingly consumed most of the offered feed. Some pigs had small amounts of feed refusal that were recovered, dried, and weighed. 

### 3.1. Performance Results

Table 2 includes the effect of the dilution ratio on the mean values of total weight gain (kg) and ADFI (g/d) of the pigs measured during the first and the second trials, respectively. 

During trial 1, pigs on the 0.6:1 dilution rate had a 25.9% higher weight gain (*p* < 0.01) than those on CON. During trial 2, pigs on 1.35:1 and 2.7:1 dilution rates had a tendency (*p* = 0.064), showing 14.1% higher weight gain than those on CON. Average daily feed intake was unaffected by the water-to-feed ratio. 

**Figure 1:**
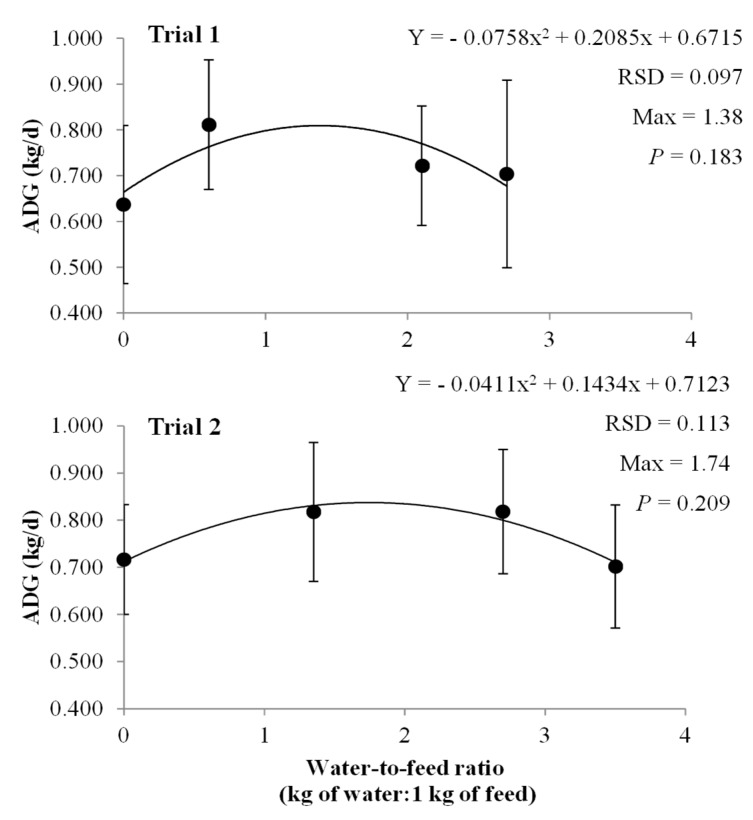
Quadratic regression for ADG in trials 1 and 2 depending on the dilution ratio. (ADG: Average daily gain; RSD: Residual standard deviation).

Figure 1 shows the same effect on the ADG (kg/d) in both experimental trials. The effect of the water-to-feed ratio on the ADG followed a quadratic evolution, with both trials showing a maximum ADG when the diet dilution rates were 1.38:1 and 1.74:1 for trials 1 and 2, respectively (Figure 1). 

Nevertheless, the *p*-values of those equations were not significant (around 0.20).

### 3.2. Digestibility 

The effects of the dilution rate on the apparent total tract digestibility coefficients are presented in Table 3. 

In trial 1, pigs fed diets with a water-to-feed ratio of 2.1:1 and 2.7:1 showed a better OMd and GEd than those on CON (*p* < 0.05). The 0.6:1 dilution ratio registered non-significant different intermediate values.

In trial 2, pigs fed diets with a water-to-feed ratio of 1.35:1, 2.7:1 and 3.5:1 showed a significantly better OMd and GEd than those on CON (*p* < 0.05). Compared to CON, the water-to-feed ratio of 3.5:1 also showed a higher (*p* < 0.05) digestibility for CP, EE and CF, and the 1.35:1 and 2.7:1 dilution for CF also. 

The regression parameters of the quadratic equations (R^2^ ≥ 0.45, *p* < 0.05) established between dilution rates and the OMd or the GEd (Table 4) showed that the highest digestibility values (the maximum of the equations) were obtained when the dilution rates were 1.83 and 2.70 or 2.72 for the first and second trial, respectively.

## 4. Discussion

Data of pig performance using different mixtures of feed and water are quite variable and often contradictory [12]. The main reasons for this seem to be related to the use of different raw materials, additives or by-products, the age range of the animals, the technological system used or the management conditions of the animals [12]. De Lange et al. [13] conclude that, based on growth performance of high-health-status pigs, there is no apparent benefit of liquid feeding growing–finishing pigs and starter pigs that are fed corn-based diets. This is in contrast to Hurst et al. [14], where swine liquid feeding is focused on wheat and barley-based diets and an effective use of co-products.

In general, our results confirm those of several authors [14,15,16,17,18] who reported a clear improvement of growing–finishing pig performance when given water-diluted diets, compared to dry diets. Jensen and Mikkelsen [16] reviewed nine trials comparing dry and liquid feed in piglets and growing–finishing pigs, showing a clear but variable improvement in daily weight gain, with a mean of 4.4% ± 5.4%, ranging from −2.6% to 15.0%. In the current work, although it is not conclusive and uses a low number of pigs, it appears the optimal water-to-feed ratio that optimizes ADG may increase (*p* < 0.21, Figure 1) with the age of the pig. The review published by Chae [19] already mentioned that the optimal water-to-feed ratio varies with the age of the pig and the method of preparing and distributing the water-feed mixture. Commercial liquid feeding devices are recommended for pigs of more than 40 kg of BW, using 2.6:1 [20] or 2.7:1 [21] as the recommended water-to-feed ratios. For lighter pigs, those dilution ratios may be too high, too bulky and may result in lower DM intake and poorer live weight gain [22]. Moreover, when dilution ratios of water-to-feed are close to 1.5:1 or lower, some authors [19,23] call this mixture “paste feeding” (wet feeding) instead of “liquid feeding”.

Since no differences in feed intake were observed (Table 2), performance results can be explained by two reasons: Water-diluted diets improve performance by (1) reducing feed wastage [1,3,15] and/or (2) improving digestibility coefficients of the feed [8]. This is the case in the present work (see Table 3), where the water-to-feed ratio that optimizes OMd and GEd increases with the age of the pig (*p* < 0.01; Table 4). Our results are in agreement with those of Barber et al. [8] who found a similar increase in dry matter and energy digestibility by increasing the water-to-feed ratio up to 3.25:1. However, Barber et al. [8] restricted water intake to the amount included in liquid feed. Conversely, other authors [24] found no improvement, or even a decrease, in digestibility by increasing the water-to-feed ratio. These authors also found a reduction in CP and energy ileal digestibility using a T-cannula. Moreover, Pedersen and Stein [25] showed no difference in apparent digestibility of DM, GE, and phosphorus between dry feed and ratios of 1:1 and 3:1. In the present results, the effect of the water-to-feed ratio on nutrients digestibility (CPd, EEd, and CFd), which are numerically similar in the first and second trials but only reach statistical significance in the latter, suggests a highly variable digestive adaptation period between animals.

Regarding ADG, the experiment was not designed to find performance differences (due to the low number of animals used), but the numerical improvement of mixing feed with water, compared to the control diet, would be up to 0.145 kg/d and 0.125 kg/d for the ADG during trials 1 and 2, respectively. 

According to Brooks [1], mixing water with dry feed and feeding after a few minutes ensures that pigs receive a more homogeneous diet and increases the rate of hydration, especially if it is finely ground, favoring the action of both digestive and in-feed enzymes. Also, it has been postulated that the increase in diet digestibility due to an increased water-to-feed ratio could be explained by the fact that the water content of the digesta favors the digestion and absorption of nutrients. Mößeler et al. [17] indicate that liquid feeding “per se” and the DM content of the offered mixture have no effect on both the main intragastric parameters and the macroscopic evaluation of the non-glandular mucosa. However, the gastric empty rate of the liquid fraction is faster than the solid fraction [26,27]. Our results indicate that the optimal dilution rate to reach the highest digestibility coefficient and performance increases with pig age, and agrees with several authors indicating that total water consumption per unit of dry feed intake increases with the age or BW of pigs [28]. Increasing the water-to-feed ratio may limit the capacity of the young pig’s stomach [29], and excessive dilution reduces nutrient density, leading to an intake of energy and nutrients below the optimum level to fulfill the animal’s requirements [22]. 

In summary, and in the present experimental conditions, the water-to-feed ratios that optimize digestibility coefficients (organic matter and gross energy) were 1.83:1 and 2.70:1, for trial 1 (47–64 kg of BW) and trial 2 (65–86 kg of BW), respectively. The data also suggest that the recommended dilution rate to reach the optimal ADG, without modifying feed intake, may increase with pigs’ age. 

## Figures and Tables

**Table 1:** Diet composition and chemical analysis of the experimental diet (as-fed basis; g/kg).

Raw Material	g/kg
Barley	450.0
Wheat	300.0
Soya bean meal	100.7
Ground corn	63.0
Extruded rapeseed meal	60.0
Calcium carbonate	9.6
Lard	5.0
Salt	4.0
Vitamin and mineral premix ^a^	3.0
Phytase ^b^	2.0
Monocalcic phosphate	1.8
L-Lys·HCl (purity 78%)	0.5
L-Thr	0.4
Calculated analysis	g/kg
Dry matter	870.73
Ash	43.91
Crude protein (N × 6.25)	160.00
Crude fiber	41.12
Ether Extract	22.40
Gross energy (Kcal/kg)	4413
Lysine	7.50
Methionine and cysteine	5.92
Threonine	6.15
Tryptophan	1.97
Calcium	7.10
Phosphorous	4.41

^a^ The premix provided vitamins and minerals (per kg) as follows: Vitamin A, 3,000,000 IU; vitamin D_3_, 600,000 IU; vitamin E, 3644 mg; vitamin K_3_, 345 mg; vitamin B_1_, 294 mg; vitamin B_2_, 1248; pantothenic acid, 3920 mg; nicotinic acid, 8036 mg; vitamin B_6_, 686 mg; vitamin B_12_, 7 mg; choline, 25,020 mg; biotin, 16 mg; Zn, 40,052 mg as ZnO; Fe, 30,000 mg as FeSO_4_·7H_2_O; Mn, 16,554 mg as MnO; Cu, 8,000 mg as CuSO_4_·5H_2_O; I, 300 mg as Ca(IO_3_)_2_; and Se, 66 mg as Na_2_SeO_3_. ^b^ 500 FTU of Aspergillus niger (Natuphos^®^ BASF).

**Table 2:** Effect of the dilution ratio on performance in growing–finishing pigs ^1^.

Treatments ^2^	Control	0.6	1.35	2.1	2.7	3.5	SEM ^3^	*p*-Value
**Trial 1**								
Weight gain (kg)	15.53 ^a^	19.55 ^b^		17.53 ^ab^	16.97 ^ab^		0.721	0.005
ADFI (g/d) ^4^	1689.4	1796.0		1782.1	1854.8		70.78	0.433
**Trial 2**								
Weight gain (kg)	20.06		22.89		22.90	19.65	1.071	0.064
ADFI (g/d) ^4^	2240.4		2367.0		2352.4	2281.2	49.57	0.248

^1^ Mean values of performance in each water-to-feed ratio for trial 1 and trial 2. ^2^ Treatments: Control (dry diet); 0.6 (0.6:1 = 0.6 parts of water per one part of feed); 1.35 (1.35:1); 2.1 (2.1:1); 2.7 (2.7:1); 3.5 (3.5:1). ^3^ SEM: Standard error. ^4^ ADFI: Average daily feed intake. ^a,b^ Values with different letters within a row indicate a significant difference at *p* < 0.05.

**Table 3:** Effect of the dilution ratio on the coefficients of apparent total tract digestibility ^1^.

Treatments ^2^	Control	0.6	1.35	2.1	2.7	3.5	SEM ^3^	*p*-Value
**Trial 1**								
OMd	0.833 ^b^	0.857 ^ab^		0.865 ^a^	0.863 ^a^		0.671	0.019
GEd	0.802 ^b^	0.830 ^ab^		0.838 ^a^	0.837 ^a^		0.791	0.024
CPd	0.751	0.801		0.812	0.785		1.704	0.117
EEd	0.176	0.239		0.277	0.295		5.119	0.403
CFd	0.295	0.396		0.421	0.427		4.121	0.138
**Trial 2**								
OMd	0.831 ^b^		0.859 ^a^		0.858 ^a^	0.862 ^a^	0.618	0.013
GEd	0.801 ^b^		0.833 ^a^		0.837 ^a^	0.838 ^a^	0.768	0.015
CPd	0.761 ^b^		0.814 ^ab^		0.801 ^ab^	0.829 ^a^	1.432	0.032
EEd	0.191 ^b^		0.313 ^ab^		0.296 ^ab^	0.335 ^a^	3.489	0.054
CFd	0.324 ^b^		0.448 ^a^		0.452 ^a^	0.486 ^a^	2.779	0.007

^1^ Mean values of digestibility in each water-to-feed ratio for trial 1 and trial 2. ^2^ Treatments: Control (dry diet); 0.6 (0.6:1 = 0.6 parts of water per one part of feed); 1.35 (1.35:1); 2.1 (2.1:1); 2.7 (2.7:1); 3.5 (3.5:1). ^3^ SEM: Standard error. OMd: Digestibility coefficient of organic matter; GEd: Digestibility coefficient of gross energy; CPd: Digestibility coefficient of crude protein; EEd: Digestibility coefficient of ether extract; CFd: Digestibility coefficient of crude fiber. ^a,b^ Values with different letters within a row indicate a significant difference at *p* < 0.05.

**Table 4:** Regression equations showing the relation between dilution rate (X) and digestibility coefficients (Y) ^1^.

	Equations	Max ^2^	R^2^ ^3^	RSD ^4^	*p*-Value
**Trial 1**					
OMd	−1.0221x^2^ + 3.7509x + 83.437	1.83	0.53	1.327	0.008
GEd	−1.1523x^2^ + 4.2256x + 80.440	1.83	0.45	1.570	0.011
**Trial 2**					
OMd	−0.4323x^2^ + 2.3303x + 83.139	2.70	0.56	1.221	0.005
GEd	−0.5151x^2^ + 2.8011x + 80.145	2.72	0.55	1.507	0.006

^1^ Quadratic equations for OMd (digestibility coefficient of organic matter) and GEd (digestibility coefficient of gross energy) in trial 1 and trial 2; ^2^ Max: Maximum ratio of dilution where the digestibility was the highest; ^3^ R^2^: Regression coefficient of the equation; ^4^ RSD: Residual standard deviation.
